# Therapeutic Approaches for Non-Melanoma Skin Cancer: Standard of Care and Emerging Modalities

**DOI:** 10.3390/ijms25137056

**Published:** 2024-06-27

**Authors:** Stefano Sol, Fabiana Boncimino, Kristina Todorova, Sarah Elizabeth Waszyn, Anna Mandinova

**Affiliations:** 1 Cutaneous Biology Research Center, Massachusetts General Hospital and Harvard Medical School, Charlestown, MA 02129, USA; ssol@mgh.harvard.edu (S.S.); fboncimino@mgh.harvard.edu (F.B.);; 2 Drexel University College of Medicine, 60 N 36th Street, Philadelphia, PA 19104, USA; 3 Broad Institute of Harvard and MIT, 7 Cambridge Center, Cambridge, MA 02142, USA; 4 Harvard Stem Cell Institute, 7 Divinity Avenue, Cambridge, MA 02138, USA

**Keywords:** non-melanoma skin cancer, cutaneous squamous cell carcinoma, basal cell carcinoma, Merkel cell carcinoma, immunotherapy, targeted therapy, new drugs

## Abstract

Skin cancer encompasses a range of cutaneous malignancies, with non-melanoma skin cancers (NMSCs) being the most common neoplasm worldwide. Skin exposure is the leading risk factor for initiating NMSC. Ultraviolet (UV) light induces various genomic aberrations in both tumor-promoting and tumor-suppressing genes in epidermal cells. In conjunction with interactions with a changed stromal microenvironment and local immune suppression, these aberrations contribute to the occurrence and expansion of cancerous lesions. Surgical excision is still the most common treatment for these lesions; however, locally advanced or metastatic disease significantly increases the chances of morbidity or death. In recent years, numerous pharmacological targets were found through extensive research on the pathogenic mechanisms of NMSCs, leading to the development of novel treatments including Hedgehog pathway inhibitors for advanced and metastatic basal cell carcinoma (BCC) and PD-1/PD-L1 inhibitors for locally advanced cutaneous squamous cell carcinoma (cSCC) and Merkel cell carcinoma (MCC). Despite the efficacy of these new drugs, drug resistance and tolerability issues often arise with long-term treatment. Ongoing studies aim to identify alternative strategies with reduced adverse effects and increased tolerability. This review summarizes the current and emerging therapies used to treat NMSC.

## 1. Introduction

Skin cancer has the highest incidence of all forms of cancer worldwide, with approximately 90% of these cases classified as non-melanoma skin cancer (NMSC) [1]. The leading cause of NMSCs is unprotected exposure to ultraviolet (UV) radiation, although many other variables also contribute to their pathogenesis [2]. Among these factors, immune suppression from various causes significantly exacerbates the risk and aggressiveness of NMSCs. In 2020, GLOBOCAN estimated that approximately 1.2 million new cases of NMSC occur annually, though this count may be underestimated due to challenges with NMSC identification and documentation [3]. Among NMSCs, the most frequent are basal cell carcinoma (BCC) and cutaneous squamous cell carcinoma (cSCC), while Merkel cell carcinoma (MCC) is less commonly encountered [4]. While the mortality rate from these cancers is relatively low, the prognosis worsens if the cancers metastasize. The incidence of metastatic BCC is estimated to be less than 0.1%, with a median overall survival (OS) of 10 months [5]. The metastatic potential of cSCC varies from 0.3% to 3.7% with a median OS of 2.19 years [6]. cSCC and BCC have better prognoses than MCC, which has a 5-year OS rate of ≤18% [7].

Surgical excision is still the most prevalent therapeutic approach in individuals who present with NMSC [8]. Late-stage NMSC patients, however, often are not candidates for surgery and have significant rates of post-surgical recurrence if they are surgical candidates. Recent studies aim to solve this problem by developing targeted therapeutics and immunotherapy [9]. Researchers have identified dysregulated intracellular signaling pathways in NMSCs for this purpose. The hedgehog protein signaling pathway has been identified as a target for BCC, and the epidermal growth factor receptor (EGFR) has emerged as a target for cSCC. Immunotherapy studies have progressed in treating systemic disease by applying immuno-checkpoint inhibitors targeting the PD-1/PD-L1 pathway and CTLA-4. The development of treatments with the highest efficacy and fewest adverse effects will necessitate continued research for years to come. This review aims to provide a comprehensive exploration of the traditional and the latest therapeutic approaches in the treatment of NMSCs.

## 2. Pathogenesis of NMSC

NMSCs are characterized by aberrant cell growth and a high mutational burden. Their development is primarily due to exposure to ultraviolet radiation (UV), although fair skin, immunosuppression, viruses, and certain hereditary disorders also contribute to the pathogenesis of these diseases [10,11].

### 2.1. UV Radiation

The most prevalent sites of NMSC are the most sun-exposed areas of the body, such as the head and neck region, followed by the upper limbs [12,13]. A correlation between the geographical incidence of NMSC and latitudinal variation in the intensities of UV radiation has been demonstrated [12,14]. Countries with the highest risk of NMSC incidence are predominantly those with a more significant proportion of people with fair skin [1]. Individuals with darker skin tones, who have higher melanin content, are generally more protected against UV-induced damage [15]. UV radiation exerts its destructive effects through DNA damage and oxidative stress, which can subsequently lead to gene mutations and inflammatory responses [16,17]. The dysregulation of diverse signaling pathways caused by UV damage has been shown to impair the ability to metabolize free radicals and to promote abnormal keratinocyte mitosis. These changes may contribute to the development of NMSC [18].

### 2.2. Immunosuppression

Individuals with impaired immune systems are at a relatively increased risk of developing cutaneous malignancies when compared to healthy individuals [11]. Immunosuppressed patient cohorts comprise solid organ transplant recipients, patients afflicted with hematologic neoplasms such as chronic lymphocytic leukemia, and individuals suffering from certain viral infectious diseases such as acquired immunodeficiency syndrome (AIDS) [19,20]. Patients who have undergone transplantation and thus have been treated with prolonged immunosuppressive therapy exhibit an increased risk of developing skin malignancies [21]. Among the NMSC, the incidence of cSCC in immunosuppressed patients is the most prominent [22,23]. Furthermore, tumors exhibit more aggressive behavior in transplant recipients, with a higher risk of metastasis and mortality [24]. 

### 2.3. Oncogenic and Tumor Suppressive Pathway

Compared to all other types of cancer, NMSCs are associated with a significantly elevated tumor mutational burden (TMB). The median number of mutations per megabase (Mb) is 45.2, 47.3, and 53.9 in cSCC, BCC, and MCC, respectively [25,26]. The high TMB is thought to be related to prolonged exposure to UV radiation. The mutational landscape of NMSC typically results in the impairment of signal transduction pathways involved in cell cycle control and the regulation of differentiation [27,28]. 

Various mutations are detected in cSCC, most commonly in the *TP53*, *NOTCH1*, *NOTCH2*, *CDKN2A*, *HRAS*, and *EGFR* genes [27,29]. Mutations in the tumor suppressor p53 can lead to alterations in the apoptosis of cells with damaged DNA and uncontrolled proliferation of keratinocytes [30,31]. Meanwhile, mutations inhibiting NOTCH signaling, often in combination with RAS and PI3K mutations, are primarily associated with the dysregulation of keratinocyte differentiation [32,33]. Targeted therapeutic strategies mainly focus on inhibiting the EGFR, which is frequently mutated or deregulated in cSCC [34]. Its dysregulation leads to downstream activation of signaling pathways, including Ras-Raf-MEK-ERK and PI3K, which affect keratinocyte proliferation and differentiation [34]. 

The Hedgehog (Hh) signaling pathway is central to BCC initiation and progression [35]. Dysregulated activation of the Hh signaling pathway is mainly due to Patched 1 (PTCH1) loss-of-function mutations and G-protein-coupled smoothed (SMO) transmembrane receptor activating mutations [36]. PTCH1 is a constitutive inhibitor of SMO. In the presence of the ligand Sonic Hedgehog, SMO is no longer repressed by PTCH1, activating the SMO-SUFU-GLI cascade. Glioma-associated (GLI) transcription factors are free to translocate to the nucleus, where they regulate the transcription of several genes that control cell proliferation and growth [37].

By contrast, two pathways are thought to contribute to MCC pathogenesis: UV radiation and Merkel cell polyomavirus (MPyV) [38]. The development of MCC is more often associated with MPyV [39]. Virus-positive (VP)-MCC accounts for almost 80% of the MCCs in the USA, while the incidence of virus-negative (VN)-MCC increases in areas with high UV exposure and fair-skinned populations [40,41]. It is important to note that VP- and VN-MCC show significant differences in mutational burden and genomic stability, with TP53 and RB1 being the most frequently mutated genes in VN-MCC [26,42]. Meanwhile, small T antigen (sT) and the truncated form of large T antigen (LT), which are expressed upon the integration of MPyV DNA into the host cells, are critical drivers for carcinogenesis in VP-MCC [43]. The essential function of truncated LT in VP-MCC is to inhibit Rb and p53, which, similarly to VN-MCC, leads to uncontrolled MCC cell proliferation [44,45].

## 3. Cutaneous Squamous Cell Carcinoma

### 3.1. Clinical Features and Staging of cSCC

cSCC often appears as a non-healing ulcer or an irregular growth on sun-exposed body parts, such as the head, neck, lower lip, scalp, and forehead. It typically manifests as nodules or plaques characterized by varying degrees of ulceration, crusting, and scaling [46]. The anatomical location and subtype impact the highly variable clinical characteristics of cSCC [47,48]. These subtypes differ significantly in their biological behavior, with some showing a strong predisposition for aggressive and often metastatic activity and others being relatively indolent. Several cSCC subtypes can be categorized as low-, intermediate-, or high-risk variations based on the likelihood of metastasis and tumor recurrence [49]. cSCC in situ lesions are the earliest form of squamous cell skin cancer. They usually grow slowly and present with no symptoms [46]. These kinds of lesions can vary in appearance from a tiny keratotic papule to a scaly pink area. A clearly defined, erythematous, red plaque is the hallmark of Bowen disease, a form of in situ cSCC [50]. Invasive cSCC may develop from up to 5% of in situ cSCC. Histologic perineural invasion is seen in lesions of invasive cSCC, particularly in high-risk cases [51].

cSCC presents a challenge in identifying high-risk cases, complicating treatment strategies and prognosis assessment. Other factors, such as nodal involvement and in-transit metastases, further add to the complexity of prognosis determination [52,53]. Brigham and Women’s Hospital (BWH) (Boston, MA, USA) has identified four key risk factors that improve predictive accuracy compared to traditional staging systems like the American Joint Committee on Cancer (AJCC) and Union for International Cancer Control (UICC) [54]. These include tumor diameter ≥ 2 cm (linked to local recurrence), invasion beyond subcutaneous fat (associated with nodal metastasis risk), poor differentiation status, and perineural invasion (the latter two both being related to disease-specific death risk). The National Comprehensive Cancer Network (NCCN) defines high-risk cSCC based on various clinical and histological factors, including tumor size, margin definition, recurrence, and histological characteristics like poor differentiation [55]. The NCCN mainly focuses on treatment guidance, while the BWH prioritizes prognostic assessment, and the AJCC categorizes stages from T0 to T4 based on tumor size and depth of invasion [56]. Recent studies have demonstrated that BWH predicts metastasis and mortality more effectively than AJCC [57]. This is due to its higher specificity and positive predictive value. This classification framework assists clinicians in devising treatment plans and forecasting patient outcomes [49,58].

### 3.2. Current Available Therapies

cSCC is predominantly managed through surgical excision and histopathologic scrutiny, achieving remarkable cure rates of up to 95% [4]. Initial management of early-stage cSCC predominantly relies on surgical resection, boasting a meager recurrence rate of approximately 4.6% and negligible metastatic potential [59,60]. High-risk cSCCs are best managed with micrographically controlled surgery (MCS), which minimizes the removal of uninvolved tissue and provides a thorough margin inspection [61].

In scenarios where surgery is not indicated, radiation therapy emerges as a viable alternative. Radiotherapy offers compelling outcomes, showcasing high cure rates particularly in early-stage cSCC [62,63]. Although these standard-of-care methods are effective, they are often invasive and may lead to considerable scarring and fibrosis. Alternatively, the management of cSCC includes various physical treatment options such as laser therapies, cryotherapy, curettage, and photodynamic therapy [64,65,66]. Among these, photodynamic therapy (PDT) utilizes a photosensitizer along with light exposure in an oxygen-rich environment to eliminate cancer cells. Over the past twenty years, PDT has developed into an effective treatment method for NMSCs including superficial and thin nodular BBC and in situ cSCC.

These modalities are highly effective for low-risk, localized cSCC, especially when conventional surgical intervention is not an option. Despite their effectiveness and favorable aesthetic outcomes, these modalities lack histological assessment capabilities. Advanced cSCC, typified by stage III or higher tumors, poses therapeutic challenges that extend beyond conventional surgical or radiotherapeutic interventions.

**Figure 1:**
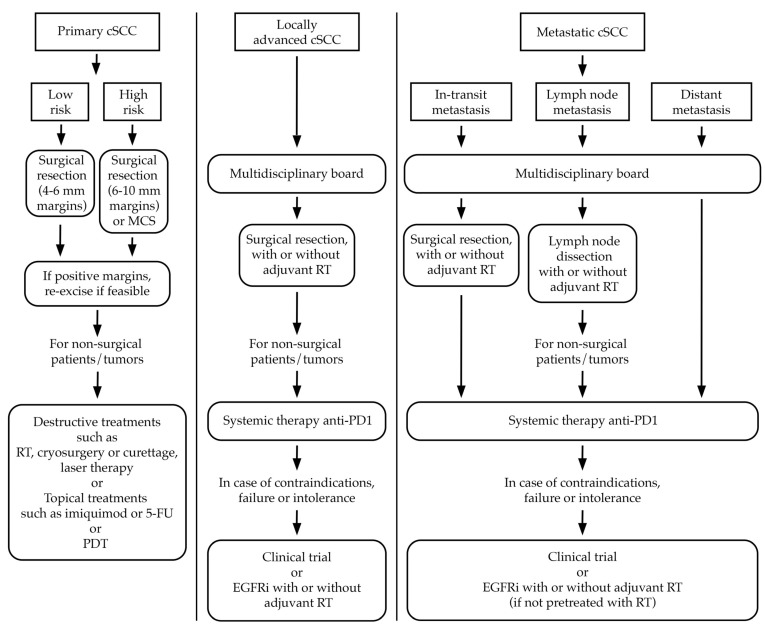
Simplified algorithm for the treatment of cSCC. cSCC, cutaneous squamous cell carcinoma; MCS, micrographically controlled surgery; RT, radiotherapy; 5-FU, 5-fluorouracil; PDT, photodynamic therapy.

Figure 1 illustrates the algorithm for the treatment of cSCC.

#### 3.2.1. Topical Treatment

For patients who are not candidates for surgery, topical treatments such as imiquimod or 5-fluorouracil (5-FU) creams offer alternative approaches to manage precancerous lesions or localized cSCC. Topical 5-FU disrupts intracellular nucleotide pools, selectively targeting rapidly proliferating cells in abnormal skin, leading to inflammation, erosion, and lesion resolution. Its targeted cytotoxicity makes it a promising choice in dermato-oncology, effectively treating malignant and non-malignant skin conditions while minimizing harm to healthy skin cells [67,68,69,70,71]. Imiquimod acts as a Toll-like receptor 7 agonist and serves as a topical immunostimulatory agent. Treatment with imiquimod has achieved remission rates exceeding 70% in patients with localized cSCC, although surgery is still considered more effective overall [72]. However, imiquimod has not been shown to be effective in treating or preventing cSCC in immunocompetent patients [73]. Recent advances in topical immune-modulating treatments include the utilization of thymic stromal lymphopoietin (TSLP). Known for its strong antitumor properties in skin with compromised barriers, TSLP has shown considerable promise in enhancing skin cancer therapies [74]. It can be activated in the skin by calcipotriol (calcipotriene), a topical medication approved by the FDA for treating psoriasis [75]. A recent randomized, double-blind clinical trial with immunocompetent patients found that combining calcipotriol with 5-FU not only eradicated actinic keratoses (precursor lesions of cSCC) but also triggered a robust immune response involving CD4+ and CD8+ tissue-resident memory cells targeting premalignant epidermal clones. Three years later, the trial revealed that significantly fewer individuals in the calcipotriol plus 5-FU treatment group developed new cSCC cases compared to those in the control group [76,77].

#### 3.2.2. Targeted Therapy

In the past, advanced cSCC was predominantly treated through targeted therapeutic strategies, particularly emphasizing the utilization of EGFR inhibitors [78,79]. However, with the advent of immunotherapy, the role of these agents has become less prominent. Among the most promising therapeutic agents are cetuximab and panitumumab.

The administration of cetuximab, whether independently or in combination with radiotherapy or platinum-based agents, has demonstrated clinical effectiveness in managing advanced cSCC. Phase II trials have investigated EGFR inhibitors, such as cetuximab, in patients with metastatic or locally advanced cSCC, displaying variable efficacy [80,81]. The objective response rate (ORR) ranged from 28% to 42%. Conversely, when used as a standalone treatment for cSCC, panitumumab has shown notable safety and efficacy profiles. Cetuximab is often utilized as a secondary-line option following primary or acquired resistance to a PD1 therapy, while panitumumab awaits formal approval for the treatment of cSCC despite demonstrating efficacy [82]. Genetic studies show a low prevalence of mutations in RAS, BRAF, and EGFR in cSCC, indicating that pre-screening before cetuximab therapy may not be necessary [83]. However, caution is recommended due to the potential for adverse events associated with cetuximab and panitumumab therapies, including infections and bleeding related to EGFR-targeted therapies.

#### 3.2.3. Systemic Immunotherapy

Systemic immunotherapy has revolutionized the treatment landscape for advanced or metastatic non-melanoma skin cancers like cSCC, especially for patients who are unsuitable candidates for surgery or radiation therapy. Checkpoint inhibitors such as cemiplimab and pembrolizumab have emerged as key players, targeting pathways like PD-1/PD-L1 and significantly improving patient outcomes. The advent of immunotherapy has transformed the management of advanced non-melanoma skin cancers, offering new hope for patients previously facing limited treatment options and poor outcomes.

Cemiplimab, a high-affinity monoclonal antibody against PD-1, has gained regulatory approval from both the United States Food and Drug Administration (FDA) and the European Medicines Agency (EMA) for managing advanced cSCC and locally advanced BCC, administered intravenously every three weeks at a dosage of 350 mg per infusion [84,85,86,87]. Clinical trials, including the landmark EMPOWER-CSCC-1 trial (n = 78), have demonstrated efficacy across metastatic and locally advanced cSCC cohorts with an ORR of 47%, CR rate of 6.7%, and tolerability, irrespective of PD-L1 expression status [86,87,88,89]. In regard to its safety, cemiplimab exhibits a toxicity profile comparable to that of other PD-1/PD-L1 inhibitors. Grade 3 or higher immune-related adverse effects (irAEs) were reported in 10% of patients, with the most common being pneumonitis (3%), diarrhea (2%), and fatigue (2%) [88]. Real-world data reaffirm cemiplimab’s effectiveness and safety, establishing it as a frontline option for unresectable or metastatic cSCC [89,90,91,92,93]. Neoadjuvant cemiplimab therapy has shown promising outcomes, achieving pathological remission in a significant proportion of patients before surgical excision.

Pembrolizumab, a humanized PD-1 blocking antibody, has emerged as a significant therapeutic option in managing cSCC, particularly for cases resistant to conventional therapies [94]. Pembrolizumab received FDA approval as a first-line treatment for locally advanced unresectable or metastatic cSCC based on the KEYNOTE-629 trial (n = 105), in which the patients were administered intravenously every three weeks at the dosage of 200 mg for up to 35 cycles or until progression [95,96]. Pembrolizumab achieved an overall ORR of 40.3% and CR rate of 12.6% in all populations, with an ORR of 50% in the locally advanced cohort and an ORR of 35.2% in the recurrent/metastatic group. Other studies, such as the CARSKIN trial (n = 57), further supported pembrolizumab’s efficacy as a first-line agent for unresectable cSCC, reporting an ORR of 41% at week 15 and a CR rate of 21% [97]. In conclusion, pembrolizumab demonstrates both efficacy and tolerability in the treatment of cSCC, showing promise in recurrent or metastatic settings and as a first-line therapy. A total of 11.9% of patients experienced Grade 3–5 immune-related adverse events (irAEs), including severe skin reactions, immune colitis, and immune hepatitis [96]. Tragically, two fatalities occurred due to treatment-related adverse events: one from cranial nerve neuropathy and another from immune-mediated colitis.

### 3.3. New Treatment Modalities

In addition to cetuximab and panitumumab, other EGFR inhibitors were explored in phase II studies, such as gefitinib (n = 40), erlotinib (n = 29), and lapatinib (n = 10). Gefitinib treatment of patients with recurrent and metastatic cSCC was conducted in a phase II study (NCT00054691) at The University of Texas M. D. Anderson Cancer Center. The treatment regimen consisted of administering gefitinib orally at a dose of 250 mg per day until either disease progression or the onset of intolerable side effects, showing an ORR of 16% [98]. In a phase 2 trial (NCT01198028), the efficacy of erlotinib was evaluated in patients with recurrent or metastatic cSCC who were unsuitable for curative treatment [99]. Thirty-nine patients received 150 mg of erlotinib daily. The overall response rate was 10%, and no unexpected toxicities were observed. A phase 2 clinical trial (NCT0166431) was conducted to investigate the impact of lapatinib on cutaneous squamous cell carcinoma (cSCC) and precursor lesions. Ten male patients received neoadjuvant lapatinib therapy, demonstrating a reduction in cSCC size in two patients and a 30% decrease in precursor lesions after 56 days [100]. These findings suggest potential for further research, particularly in high-risk patients.

The involvement of the mTOR pathway in cSCC and other NMSCs has attracted significant interest as well [101]. mTOR inhibitors have demonstrated potential as efficacious treatments, particularly in solid-organ transplant recipients. Topical rapamycin treatment has shown efficacy in conditions such as Paget’s disease and extramammary Paget’s disease (EMPD) [102,103,104]. In conclusion, while EGFR inhibitors remain pivotal, targeted therapies and mTOR inhibitors offer promising alternatives.

Two promising new PD-1 inhibitors effective in treating advanced and metastatic cSCC are nivolumab and cosibelimab. Nivolumab has also been investigated in the first-line treatment of advanced CSCC and in the second-line therapeutic option for head and neck cSCC in China [105]. A phase II investigation (NCT03834233) in which the patients were administered intravenously every two weeks at a dosage of 3 mg/kg for up to 12 months revealed encouraging results, with an ORR of 54.5% at 24 weeks for advanced cSCC, albeit accompanied by notable treatment-related AEs affecting 21% of patients (n = 24) [106]. Cosibelimab was studied in a phase I trial (NCT03212404) in patients with metastatic cSCC (n = 10) [107]. Administered intravenously at 800 mg every two weeks, it yielded an ORR of 47.4% among participants, suggesting potential as a therapy for metastatic cSCC with a tolerable safety profile. The most common adverse events included fatigue, rash, and anemia, with manageable immune-related adverse events reported in 23.1% of participants.

In addition to checkpoint inhibitors, alternative immunotherapeutic approaches such as intralesional injections of talimogene laherparepvec (T-VEC), an HSV-1 oncolytic virus FDA-approved for the local treatment of unresectable recurrent melanoma, are being investigated (NCT03714828). Clinical trials are exploring combination therapies to enhance response rates and improve patient prognosis [108,109]. In this pilot study, all seven participants achieved impressive ORR and CR values of 100%, with mild adverse effects. These findings highlight TVEC’s potential as a treatment option for unresectable cSCC (Table 1).

## 4. Basal Cell Carcinoma

### 4.1. Clinical Features and Staging of BCC

BCC is the most common form of human skin cancer arising from the basal layer of the epidermis [110]. It is more commonly observed in sun-exposed areas of the skin, including the face, neck, and trunk [111,112,113,114]. BCCs are typically small or intermediate-size lesions that progress slowly and display benign clinical prognosis. In some cases, local tissue infiltration and ulceration can occur, although BCC is rarely associated with the onset of metastasis [115]. Metastatic BCC and locally advanced BCC are distinguished by marked invasiveness, rapid growth rate, and tendency to recur [116,117]. These types of BCC often result in a poor prognosis [118]. The clinical features of BCC are diverse, and several variants have been described. However, BCC is typically classified into three categories, superficial, nodular, and infiltrative, with the last one being the least common and characterized by superficial blood vessels and local tissue invasion [110,119].

According to the NCCN, BCC staging is based on the likelihood of recurrence, which varies depending on the location of the tumor. Thus, low-risk, high-risk, and regional or distant metastasis are categorized into three categories. Low-risk BCCs are minor and superficial with clear and defined edges, while high-risk BCCs measure at least 2 cm in width and have returned despite therapy in the past. If a lesion has spread to a remote site, it is classified as regional or distant metastatic BCC. A novel classification released by the European Association of Dermato-Oncology (EADO) that considers factors including size, location, boundary definition, previous treatments, and related recurrences divided BCCs into two categories: easy-to-treat BCC and difficult-to-treat BCC [120,121,122]. Easy-to-treat BCC accounts for about 95% of BCCs with low recurrence risk. In comparison, difficult-to-treat BCC includes locally advanced and metastatic BCC, which have an estimated incidence of 0.8% and 0.0028–0.55%, respectively [123,124]. This classification is essential for determining the most appropriate treatment approach [114].

### 4.2. Current Available Therapies

Surgery is the primary treatment modality for easy-to-treat BCCs, which also offers the opportunity to perform a deeper examination of the safety margins and histological characteristics of the tumor [120,125,126]. MCS should be instead utilized in patients with high-risk BCC. However, it should be noted that surgery is associated with a high risk of relapse. Radiotherapy has been identified as a potential treatment alternative for patients who are not eligible for surgical intervention, with a comparable recurrence rate to that of surgery [127]. When surgery and radiotherapy are unsuitable or contraindicated for the patient, second-line treatment options may include topical treatment, targeted Hh inhibitors, or PD-1 inhibitor immunotherapy. 

**Figure 2:**
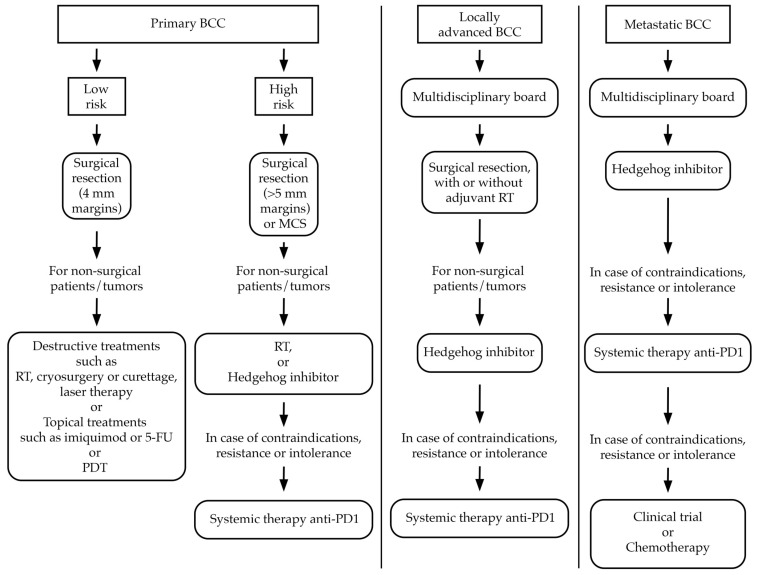
Simplified algorithm for the treatment of BCC. BCC, basal cell carcinoma; MCS, micrographically controlled surgery; RT, radiotherapy; 5-FU, 5-fluorouracil; PDT, photodynamic therapy.

Figure 2 illustrates the algorithm for the treatment of BCC.

#### 4.2.1. Topical Treatment

Topical treatments are indicated as an option in low-risk superficial BCCs [128]. Topical agents exhibit fewer side effects, are more cost-effective, and have lower cosmetic implications, especially in patients where multiple BCC lesions necessitate multiple excisions [129,130,131,132]. Imiquimod 5% and 5-fluorouracil 5% cream are currently FDA-authorized for treating small superficial BCCs [133,134,135]. Imiquimod is an immune response modifier that stimulates the release of inflammatory immunomodulatory cytokines, leading to apoptosis of BCC [136]. The treatment is indicated for use in immunocompetent adults and requires the application of the cream five times per week for six weeks. Fluorouracil is a chemotherapeutic agent that acts as a pyrimidine antimetabolite, interfering with DNA synthesis and inhibiting the proliferation of BCC cells [137,138]. It must be applied twice daily for two to four weeks [135]. The most common adverse effects are localized skin irritations, including burning and itching. The immune-modulating effect of imiquimod may be more effective in preventing superficial BCC recurrence [139,140]. The concerns regarding the utilization of topical treatment for BCC are related to the difficulties of self-application by patients, the potential adverse reactions, and the restricted application limited to low-grade superficial lesions.

#### 4.2.2. Hedgehog Inhibitors

Aberrant activation of the Hh pathway and the subsequent loss of SMO receptor inhibition is a hallmark of basal cell carcinoma [141]. The loss of SMO inhibition induces a cascade activation that promotes tumor cell division and proliferation [142,143]. Identifying the role of Hh pathway mutations in BCC pathogenesis pioneered the development of Hh inhibitor treatment. Two oral Hh inhibitors, vismodegib and sonidegib, have been FDA-approved for patients with locally advanced BCC and metastatic BCC [144]. These drugs are semi-synthetic derivatives of natural alkaloid cyclopamine and target SMO protein [145]. Oral administration is convenient and preferred by patients due to non-invasiveness. However, adverse side effects, including muscle spasms and alopecia, may be alleviated with topical therapy [146,147]. Although Hh inhibitors are an effective alternative for treating advanced BCC, drug resistance is one of the significant concerns, and known SMO mutations can confer functional resistance to vismodegib and sonidegib [148,149]. Vismodegib is approved worldwide for treating adult patients with locally advanced BCC or metastatic BCC who are not candidates for surgery or radiotherapy [150,151]. It was the first authorized Hh inhibitor following the success of the phase I ERIVANCE trial in which patients with locally advanced BCC (n = 15) or metastatic BCC (n = 18) received 150 mg daily. The ORR was 43% and 30%, respectively, and the results were in line with subsequent studies like the STEVIE trial, which enrolled 1215 subjects with an ORR of 69% in the locally advanced BCC group and 37% in patients with metastatic BCC [152]. However, the common adverse effects such as muscle spasms, alopecia, fatigue, and nausea have a high incidence and often result in treatment discontinuation [153]. In addition, the rate of intrinsic or acquired resistance to vismodegib is as high as 20% within the first year of treatment [154]. At a daily dose of 200 mg, sonidegib is the second FDA-approved drug for treating unresectable locally advanced BCC [155,156]. In fact, in the BOLT trial of patients with locally advanced BCC and metastatic BCC receiving sonidegib at 800 mg and 200 mg daily, tumor response was independent of sonidegib dose in this range, suggesting 200 mg/day instead of 800 mg/day [157]. The final report at 42 months showed that ORRs were 56% for locally advanced BCC and 8% for metastatic BCC in the 200 mg group and 46.1% and 17%, respectively, in the 800 mg cohort [158]. The most common adverse effects were similar to those reported with vismodegib but also included raised creatinine and lipase concentration [157].

#### 4.2.3. Systemic Immunotherapy 

BCC cells express PD-L1, which, through the binding of the PD-1, can lead to the inhibition of T cell function [159,160]. Cemiplimab is an anti-PD-1 monoclonal antibody that enhances antitumor responses. It has been FDA-approved for treating locally advanced BCC and metastatic BCC in patients with progressive disease, resistance or intolerance to Hh inhibitors [161]. In a study involving 84 locally advanced BCC patients, cemiplimab was administered intravenously at a dosage of 350 mg every three weeks for up to 93 weeks. The ORR was 31%, including 6% CR and 25% PR [162]. However, 97% of participants had adverse effects, most commonly fatigue and itching, but colitis and adrenal insufficiency were observed [162].

### 4.3. New Treatment Modalities

New clinical trials are underway for BCC, some of which have reported successful preliminary results. Patidegib is a novel SMO inhibitor under development for topical and oral administration. In a study involving 36 subjects with nodular BCC, patients were treated topically once or twice daily with patidegib 2% or 4% gel for a total of 12 weeks, with patidegib 2% gel showing a greater efficacy and lower adverse effects (NCT02828111). Meanwhile, oral administration of patidegib at various doses (20 to 210 mg/day) was associated with the most commonly reported adverse effects of muscle spasms, fatigue, nausea, and hair loss in a phase I study involving 94 patients with advanced BCC [163]. Other studies are ongoing, and promising outcomes have been evaluated, evaluating the efficacy of oral administration of second-generation Hh inhibitors such as taladegib and itraconazole (NCT03972748) [164,165,166]. Topical itraconazole treatment is assessed in two phase I trials (NCT02735356). Regarding immunotherapy, there has also been progress in clinical trials on PD-1 inhibitors. Recent studies have investigated the neoadjuvant use of oral pembrolizumab (NCT02690948) and nivolumab (NCT03521830) (Table 2).

## 5. Merkel Cell Carcinoma

### 5.1. Clinical Features and Staging of MCC

MCC is a rare and aggressive skin cancer with two main types: the virus-negative MCC (VN-MCC), driven by ultraviolet-induced mutations, and virus-positive MCC (VP-MCC), driven by Merkel cell polyomavirus (MPyV) [38,167]. Clinically, MCC presents as painless but rapidly expanding flesh-colored nodules, mainly in the skin or subcutaneous tissue [168]. Usually, MCC lesions are on sun-exposed skin, most commonly on the extremities, head, and neck. MCCs are often confused with other lesions like cysts or melanomas. Diagnosis requires biopsy due to nonspecific clinical features. However, 89% of patients with MCC show at least three of these features captured in the acronym AEIOU: asymptomatic, expanding rapidly, immunosuppression, older than age 50, and UV radiation [169]. Confirmation involves immunohistochemistry for neuroendocrine and epithelial markers such as cytokeratin 20. MCCs can metastasize without a primary lesion.

The 8th Edition AJCC staging system for MCC refines staging by distinguishing between clinical and pathological assessments using TNM criteria [170]. The majority of patients exhibit local (65%), nodal (26%), or distant (8%) disease. Survival outcomes are better for patients with pathological staging data. An extensive evaluation, encompassing physical examination and imaging, is vital for precise staging. If metastasis is not identified, patients may undergo a sentinel lymph node biopsy (SLNB) in conjunction with primary tumor surgery [171,172]. This approach facilitates the discussion of prognosis and treatment planning for patients with MCC.

### 5.2. Current Available Therapies

Surgical excision remains the primary treatment for resectable MCC. The standard procedure involves excising the primary tumor with 1 cm lateral margins, encompassing the underlying fascia, followed by a potential SLNB [173,174]. Adjuvant radiation therapy (50–55 Grays) is recommended post-surgery, also in patients with pathologically negative lymph nodes. For patients who are unsuitable candidates for surgery or have primary tumors in anatomical locations where excision would cause notable functional limitations, radiotherapy monotherapy serves as an alternative [175,176]. A positive SLNB without distant metastasis may result in complete lymph node dissection (CLND), radiation therapy, or both [174]. Treatment options encompass radiotherapy, immunotherapy, or chemotherapy in metastasis to additional organs.

**Figure 3:**
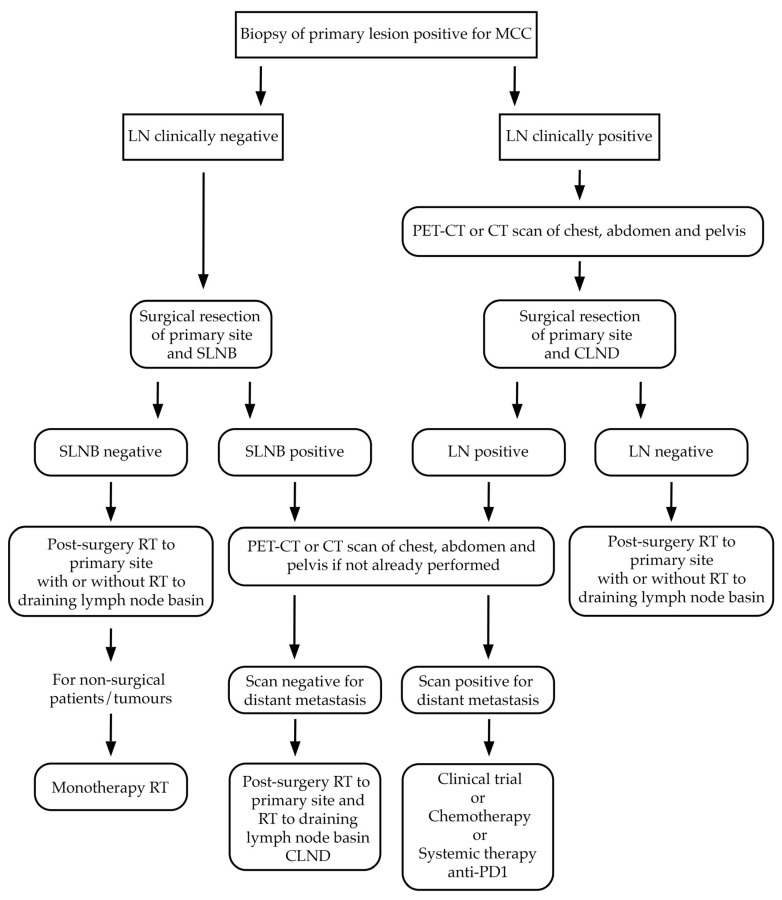
Simplified algorithm for the treatment of MCC. MCC, Merkel cell carcinoma; LN, lymph node; SLNB, sentinel lymph node biopsy; CLND complete lymph node dissection; RT, radiotherapy; PET, positron emission tomography; CT, computed tomography.

Figure 3 illustrates the algorithm for the treatment of MCC.

#### Systemic Immunotherapy

The advent of checkpoint inhibitors targeting the PD-1/PD-L1 pathway has revolutionized the treatment of metastatic MCC, now recommended as the first-line therapy by global guidelines. Avelumab, the first systemic therapy for MCC, proved effective in both second-line (n = 88) and first-line (n = 116) treatments in the JAVELIN Merkel 200 trial [7,177]. Administered intravenously every two weeks at a dosage of 10 mg/kg until treatment progression, it achieved an ORR of 33% and CR of 11% post-chemotherapy failure. In first-line treatment, a 40% ORR was observed. While PD-L1 positivity correlated with better responses, it was not a requisite for the treatment. Although not approved for the treatment of locally advanced disease alone, the off-label use of this treatment is being explored [178,179]. Pembrolizumab gained FDA approval in 2018 for recurrent locally advanced or metastatic MCC. In Keynote 017 (n = 50), in which the patients were administered intravenously every three weeks at a 2 mg/kg dosage for up to 2 years, the ORR was 58%, including 24% demonstrating an imaging CR [180,181]. Retifanlimab, another PD-1 receptor monoclonal antibody, was investigated in the POD1UM-201 phase II trial (NCT03599713), in which 87 treatment-naïve patients received 500 mg IV every four weeks for up to 2 years, with an ORR of 46% and CR of 12%. Subsequently, the FDA approved retifanlimab for adult patients with metastatic or recurrent locally advanced MC.

### 5.3. New Treatment Modalities

Targeted therapies represent a promising avenue for patients with advanced MCC who are ineligible for or unresponsive to immune checkpoint inhibitors. Merkel cell carcinoma exhibits increased angiogenesis driven by high VEGF-A expression. Trials with tyrosine kinase inhibitors (TKIs), such as pazopanib and cabozantinib, targeting VEGFR and other pathways, showed modest efficacy [182]. The use of TKIs in combination with immunotherapies is currently being investigated to improve outcomes (NCT04869137). YM155 and ABT-263, small-molecule inhibitors of antiapoptotic protein surviving and apoptosis regulator BCL-2 family proteins, have shown efficacy in inducing cell death in MCC cell lines and xenografts [183,184]. VN-MCC is frequently associated with p53 mutations, whereas VP-MCC is typically characterized by wild-type p53 [185,186]. KRT-232 (navtemadlin), an MDM2 inhibitor, demonstrated efficacy in phase 1b/2 trials (NCT03787602) for p53 wild-type MCC patients, particularly those resistant to anti-PD1/PD-L1 therapy. Despite these promising results, drug development for MCC was discontinued.

Merkel cell carcinoma expresses somatostatin receptors (SSTRs), suggesting potential avenues for treatment with somatostatin analogs such as octreotide and lanreotide [187,188]. Although the effectiveness of these treatments in MCC is limited, ongoing investigations aim to enhance outcomes by developing higher-affinity analogs and peptide receptor radionuclide therapy.

The PI3K/AKT/mTOR signaling pathway is crucial in promoting tumor cell proliferation and survival, including in the MCC [189]. MCC often exhibits elevated levels of phosphorylated AKT and occasional mutations in the PIK3CA gene, indicating pathway activation [190,191]. Inhibition of mTOR has shown promise in inducing cell death and suppressing MCC growth in preclinical models [192,193]. Despite setbacks in clinical trials targeting both mTOR complexes, recent research continues to highlight the importance of this pathway in MCC, with upregulated gene expression linked to poor outcomes [194]. Clinical responses to PI3K and AKT inhibitors underscore the therapeutic potential of targeting this pathway. There has only been a single reported case of a patient with advanced MCC carrying a known PI3K mutation successfully treated with idelalisib, a PI3K-δ inhibitor, resulting in a rapid and complete remission [195]. A clinical trial is investigating the safety and efficacy of mTOR inhibition in patients with MCC (NCT02514824). Studies have demonstrated the effectiveness of copanlisib in inhibiting tumor growth, while combination therapies with alpelisib and the BCL-2 inhibitor navitoclax show synergistic effects [196,197]. Additionally, artesunate, known for its anti-malarial properties, exhibits cytotoxic activity in MCC, particularly in MCPyV-positive cases, suggesting a novel therapeutic avenue for further exploration [198,199].

A recent preclinical study highlighted MUC1-C as a potential therapeutic target in MCC [175,200]. This study revealed that MUC1-C interacts with MYCL, promoting MCC progression and cell survival in both VP- and VN-MCC cells [201]. This finding underscores the significance of targeting MUC1-C in MCC treatment, offering a potential avenue for pharmacological intervention to impede disease progression.

In addition to the previous PD-1/PD-L1 inhibitors, nivolumab has been investigated in treating advanced MCC, achieving an ORR of 68% (CheckMate 358 phase I/II trial, NCT02488759). Combining nivolumab with ipilimumab, targeting cytotoxic T-lymphocyte associated antigen-4 (CTLA-4), enhances antitumor responses, offering potential for patients unresponsive to PD-1 blockade [202,203]. CTLA-4 is an immune checkpoint protein able to repress the function of activated T cells binding CD80 or CD86 on their surface [204]. Despite the challenges that remain, immune checkpoint inhibitors represent a pivotal advancement in the management of MCC, offering hope for prolonged survival and enhanced quality of life among patients with this aggressive malignancy.

Other potential treatment avenues under investigation include immunostimulatory agents such as MCPyV vaccination and talimogene laherparepvec (T-VEC). In situ vaccination with plasmid DNA has been demonstrated to be an effective treatment for advanced MCC patients who have shown resistance to PD-1/PD-L1 inhibitors. At the same time, off-label use of T-VEC showed promise in treating recurrent MCC, with reported partial or complete responses in several patients [205,206,207,208,209]. Clinical trials explore its potential in MCC and other non-melanoma cancers (NCT03458117, NCT02819843, and NCT02978625). These treatments may offer alternative options, particularly for patients unsuited for immunotherapy (Table 3).

## 6. Future Perspectives

Recent advancements in understanding the pathogenesis of cSCC, BCC, and MCC have significantly advanced therapeutic options, enhancing patients’ survival and quality of life. Crucial findings have highlighted the role of immune suppression in cSCC, the Hedgehog pathway’s critical involvement in BCC and its targeted inhibition’s success, and the MPyV’s influence in the development of VP- MCC. Systemic immunotherapies targeting PD-1 and PD-L1 have transformed the management of aggressive and metastatic NMSC. However, the etiological complexity of NMSC, involving both genetic and environmental factors, prevents a singular treatment approach. Furthermore, the incidence of NMSC has grown over time and this is correlated with growing treatment costs. Of the NMSC treatments that have been addressed in this review, topical therapy is more cost-effective than other non-surgical therapies since it requires fewer in-office procedures and reduces surgical risks such as scarring and infection [210]. However, as with surgery, the cost of treatment is influenced by the size of the tumor and the delay in diagnosis. It is evident that the future of NMSC management will increasingly rely on successful primary and secondary prevention strategies. Well-defined risk factors, such as UV exposure and immune suppression, provide unique opportunities for preventive strategies not typically available for other cancers. Recent studies have also highlighted the dynamic interactions within the tumor microenvironment, offering new perspectives on NMSC management, including therapies for metastatic cSCC and extending research into MCC and BCC. Future treatments may need to address the continuously evolving nature of cancer cells and the intricate interplay of genetic, immunological, and stromal factors. Adopting a holistic approach could revolutionize treatment strategies by focusing not only on eliminating cancer cells but also on altering the broader tumor environment to inhibit cancer progression.

## Figures and Tables

**Table 1:** Ongoing trials in cutaneous squamous cell carcinoma (cSCC).

	NCT Identifier	Study Title	Conditions	Phases	Patients
**Topical Treatment**	NCT00652080	An Open-Label Safety Study of API 31510 in a Topical Cream for in Situ Cutaneous Squamous Cell Carcinoma (SCCIS)	In situ cSCC	PHASE1/2	35
**EGFR Inhibitor**	NCT02324608	Cetuximab Before Surgery in Treating Patients With Aggressive Locally Advanced Skin Cancer	Locally advanced cSCC	NA	15
	NCT00126555	Gefitinib in Treating Patients Who Are Undergoing Surgery and/or Radiation Therapy for Locally Advanced or Recurrent Squamous Cell Skin Cancer	Locally advanced cSCC or Recurrent cSCC	PHASE2	23
	NCT01198028	Erlotinib in Treating Patients With Recurrent or Metastatic Skin Squamous Cell Carcinoma	Recurrent or Metastatic cSCC	PHASE2	42
**Immunotherapy**	NCT02883556	Study of Pembrolizumab as First Line Therapy in Patients With Unresectable Squamous Cell Carcinoma of the Skin	Unresectable cSCC	PHASE2	57
	NCT03834233	Nivolumab in Patients With Advanced Cutaneous Squamous Cell Carcinoma	Advanced cSCC	PHASE2	24
	NCT03212404	Phase 1 Study of CK-301 (Cosibelimab) as a Single Agent in Subjects With Advanced Cancers	Advanced cSCC	PHASE1	500
	NCT03714828	Study of TVEC in Patients With Cutaneous Squamous Cell Cancer	cSCC	PHASE2	11
	NCT04163952	Talimogene Laherparepvec and Panitumumab for the Treatment of Locally Advanced or Metastatic Squamous Cell Carcinoma of the Skin	Locally advanced cSCC or Recurrent cSCC	PHASE1	5
	NCT02760498	Study of REGN2810 in Patients With Advanced Cutaneous Squamous Cell Carcinoma	Advanced cSCC	PHASE2	432

**Table 2:** Ongoing trials in basal cell carcinoma (BCC).

	NCT Identifier	Study Title	Conditions	Phases	Patients
**Topical Treatment**	NCT05157763	A Study to Evaluate the Safety and Efficacy of EscharEx (EX-02) in the Treatment of Basal Cell Carcinoma	Superficial or nodular BC	PHASE1/2	32
	NCT00604890	Dose-Ranging Clinical Trial of Topical Creams Containing API 31510 for the Treatment of Superficial Basal Cell Carcinoma	Superficial BCC	PHASE1/2	186
	NCT03180528	Topical Remetinostat in Treating Patient With Cutaneous Basal Cell Cancer	BCC	PHASE2	30
**HH Inhibitors**	NCT02667574	Study Evaluating the Interest of Vismodegib as Neo-adjuvant Treatment of Basal Cell Carcinoma (BCC)	BCC	PHASE2	55
	NCT04806646	Tailored Sonidegib Schedule After Complete Response in BCC	laBCC	PHASE2	21
	NCT02690948	Pembrolizumab With or Without Vismodegib in Treating Metastatic or Unresectable Basal Cell Skin Cancer	Metastatic or unresectable BCC	PHASE1/2	16
	NCT06344052	To Assess the Safety and Efficacy of SP-002 With Vismodegib for the Treatment of Locally Advanced Basal Cell Carcinoma	Locally advanced BCC	PHASE2	80
	NCT03035188	Neoadjuvant Vismodegib in Patients With Large and/or Recurrent Resectable Basal Cell Carcinoma	BCC	PHASE2	40
	NCT03972748	Use Of Oral Itraconazole In Patients With Locally Limited Basocellular Carcinoma Of Skin.	BCC	NA	28
	NCT02735356	Topical Itraconazole in Treating Patients With Basal Cell Cancer	BCC	EARLY PHASE1	9
	NCT02828111	Clinical Trial of Patidegib Gel 2%, 4%, and Vehicle Applied Once or Twice Daily to Decrease the GLI1 Biomarker in Sporadic Nodular Basal Cell Carcinomas	Nodular BCC	PHASE2	36
	NCT01700049	Study Evaluating the Efficacy of Oral Vismodegib in Various Histologic Subtypes	BCC	PHASE2	28
**Immunotherapy**	NCT01327053	A Phase II Study of Efficacy and Safety in Patients With Locally Advanced or Metastatic Basal Cell Carcinoma	Locally advanced or metastatic BCC	PHASE2	230
	NCT04679480	Anti-PD1-antibody and Pulsed HHI for Advanced BCC	Advanced BCC	PHASE2	20
	NCT03521830	Nivolumab Alone or Plus Relatlimab or Ipilimumab for Patients With Locally-Advanced Unresectable or Metastatic Basal Cell Carcinoma	Metastatic or unresectable BCC	PHASE2	57
	NCT03132636	PD-1 in Patients With Advanced Basal Cell Carcinoma Who Experienced Progression of Disease on Hedgehog Pathway Inhibitor Therapy, or Were Intolerant of Prior Hedgehog Pathway Inhibitor Therapy	BCC	PHASE2	138

**Table 3:** Ongoing trials in Merkel cell carcinoma (MCC).

	NCT Identifier	Study Title	Conditions	Phases	Patients
**Targeted Therapies**	NCT04869137	Neoadjuvant Lenvatinib Plus Pembrolizumab in Merkel Cell Carcinoma	MCC	PHASE2	26
	NCT03787602	Navtemadlin (KRT-232) with or without Anti-PD-1/Anti-PD-L1 for the Treatment of Patients with Merkel Cell Carcinoma	MCC	PHASE1/2	115
	NCT02514824	MLN0128 in Recurrent/Metastatic Merkel Cell Carcinoma	Recurrent or metastatic MCC	PHASE1|2	9
**Immunotherapy**	NCT04291885	Immunotherapy Adjuvant Trial in Patients with Stage I-III Merkel Cell Carcinoma	MCC	PHASE2	132
	NCT05496036	Neoadjuvant PD-1 Blockade in Resectable Merkel Cell Carcinoma	MCC	PHASE2	15
	NCT06151236	Neoadjuvant Nivolumab and Relatlimab in Merkel Cell Carcinoma	MCC	PHASE2	20
	NCT03599713	A Study of INCMGA00012 in Metastatic Merkel Cell Carcinoma (POD1UM-201)	Metastatic MCC	PHASE2	107
	NCT02488759	An Investigational Immuno-therapy Study to Investigate the Safety and Effectiveness of Nivolumab, and Nivolumab Combination Therapy in Virus-associated Tumors	Advanced MCC	PHASE1/2	578
	NCT03458117	T-VEC in Non-melanoma Skin Cancer	MCC	PHASE1	26
	NCT02819843	A Study of T-VEC (Talimogene Laherparepvec) with or without Radiotherapy for Melanoma, Merkel Cell Carcinoma, or Other Solid Tumors	MCC	PHASE2	19
	NCT02978625	Talimogene Laherparepvec and Nivolumab in Treating Patients with Refractory Lymphomas or Advanced or Refractory Non-melanoma Skin Cancers	MCC	PHASE2	68
	NCT02155647	Avelumab in Participants with Merkel Cell Carcinoma (JAVELIN Merkel 200)	MCC	PHASE2	204
	NCT04393753	Domatinostat in Combination with Avelumab in Patients with Advanced Merkel Cell Carcinoma Progressing on Anti-PD-(L)1	Advanced MCC	PHASE2	19
	NCT05422781	Study to Evaluate The Safety, Tolerability and Immunogenicity of 4 mg of ITI-3000 In Patients with Polyomavirus-Positive Merkel Cell Carcinoma (MCC)	VP-MCC	PHASE1	6
